# Integrated hybrid Nested-bottled photobioreactor for enhanced mixing, mass transfer, and CO₂ fixation in *Arthrospira platensis* raceway pond cultivation systems

**DOI:** 10.1186/s13068-025-02670-1

**Published:** 2025-07-02

**Authors:** Ameer Ali Kubar, Shahid Mehmood, Michael Schagerl, Santosh Kumar, Xinjuan Hu, Feifei Zhu, Xiangru Xu, Jiheng Ni, Shuhao Huo

**Affiliations:** 1 School of Food and Biological Engineering, Jiangsu University, Zhenjiang, 212013 China; 2 School of Life Sciences, Jiangsu University, Zhenjiang, 212013 China; 3 Department of Functional and Evolutionary Ecology, University of Vienna, Djerassiplatz 1, 1030 Vienna, Austria; 4 School of Agricultural Engineering, Jiangsu University, Zhenjiang, 212013 China

**Keywords:** Microalgae, Photobioreactor, Cultivation method, CO₂ fixation, Photosynthetic efficiency

## Abstract

This study introduces a novel hybrid photobioreactor system that integrates an open raceway pond (ORWP) with a Nested-bottled photobioreactor (NB-PBR) in a closed-loop configuration to enhance microalgal biomass production and CO₂ fixation. The system facilitates continuous culture circulation, improving mass transfer and mixing efficiency while ensuring optimal light exposure and CO₂ dissolution. This design resulted in a 38% increase in dry mass (3.1 g/L) and improved mass transfer and mixing times by 16.6% and 15.3%, respectively. The optimized cultivation conditions led to a 39.9% enhancement in CO₂ fixation and an 8.7% increase in photosynthetic efficiency (Fv/Fm) compared to traditional systems. The strategic movement of poorly illuminated ORWP to the NB-PBR maximized light absorption and nutrient uptake, significantly boosting overall productivity. These findings highlight the potential of hybrid photobioreactor systems in improving microalgal growth efficiency and advancing sustainable algal cultivation for commercial applications.

## Introduction

The continued dependence of industrial and economic sectors on conventional energy sources has resulted in carbon dioxide (CO₂) emissions exceeding their natural removal rate, leading to a rising atmospheric CO₂ concentration. The global population reached 8 billion in 2022 and is projected to grow to 10 billion by 2100 [1]. This imbalance intensifies the greenhouse effect, contributing to severe environmental and ecological consequences [2]. Fossil fuels have long played a central role in modern energy production, supporting industrial growth and improving living standards [3]. However, their combustion remains a major source of CO₂ emissions, driving global climate change [4]. Saudi Arabia is among the leading CO₂ emitters, with key contributions from industries, electricity generation, transportation, and households [5, 6]. The energy sector alone accounts for over 90% of the country's CO₂ emissions from fossil fuels, while power generation globally contributes approximately one-third of total CO₂ emissions. Consequently, researchers worldwide are exploring strategies to reduce CO₂ emissions and mitigate global warming. [7, 8]. 

Microalgae are tiny photosynthetic organisms lacking vascular tissues, true leaves, roots and stems, capable of converting solar energy into chemical energy [9]. They exhibit much higher efficiency in light-to-energy conversion compared to terrestrial plants. Microalgae are usually grown photoautotrophically, where light serves as the primary energy source, and CO₂ is utilized for carbon assimilation [10]. These microorganisms absorb essential nutrients like nitrogen and phosphorus from their growth medium and convert them into lipids, food additives, and bioactive compounds through various metabolic pathways [11, 12]. Cultivating microalgae in wastewater offers environmental benefits by facilitating the removal of nitrogen compounds, phosphates, and heavy metals [13]. Microalgae utilize oxygenic photosynthesis to absorb CO₂, converting it into carbohydrates, lipids, and hydrocarbons, while releasing oxygen and supporting sustainable carbon sequestration [14]. 

Microalgae are recognized for their high photosynthetic efficiency and productivity and their potential to produce a diverse range of valuable compounds. Amongst these are high-value bioactive compounds such as pigments (e.g., beta-carotene, astaxanthin, lutein) and polyunsaturated fatty acids (PUFAs). Several factors can impact microalgae growth and productivity, including low nutrient supply, toxic pollutants, pathogens, and organic contaminants like azo dyes in wastewater, which can hinder photosynthetic activity and biomass accumulation [15, 16]. Specifically, the availability of carbon and nitrogen sources in the culture medium influences the biochemical composition of microalgae, including proteins, carbohydrates, chlorophyll, and fatty acids [17]. Additional factors such as strain selection, light availability, temperature, and pH are also critical for biomass production and bioactive compound synthesis [18, 19]. 

Microalgae provide a promising, low-cost alternative to traditional carbon capture and sequestration (CCS) methods, which are hindered by high energy demands, operational costs, and environmental concerns associated with physical CCS technologies [20, 21]. Microalgae naturally tolerate high CO₂ concentrations (they are even boosted by high CO_2_ supply) and can effectively utilize CO₂ from industrial emissions, including power plant exhaust gases. Through photosynthesis, microalgae fix carbon efficiently, similar to early biological carbon sequestration mechanisms. Additionally, many microalgal species can assimilate carbonates like sodium bicarbonate (NaHCO₃) and sodium carbonate (Na₂CO₃) for cellular growth, further enhancing their potential for sustainable carbon capture and utilization [22]. Microalgae biotechnology offers a promising solution to global challenges such as food security, energy, climate change, and waste management, by providing sustainable alternatives to land crops for nutrients, renewable energy, and value-added products [23]. Microalgae efficiently convert carbon sources into biomass, producing biofuels, pigments, pharmaceuticals, and other high-value compounds, driving advancements in cultivation technology [18, 24, 25].

Photobioreactors (PBRs) offer a controlled alternative, optimizing photosynthesis efficiency while minimizing environmental limitations. These systems function as solar receivers, where absorbed light not used for photosynthesis is converted into heat, potentially affecting culture conditions. Environmental factors such as light intensity, aeration, and nutrient availability critically affect PBR performance; suboptimal conditions result in reduced microalgal growth. Therefore, PBR designs need to be optimized to mitigate these limitations and enhance cultivation efficiency. Vertical tubular PBRs, with transparent tubes and gas sparging systems, enhance mass transfer, nutrient distribution, and gas exchange, leading to improved microalgal productivity. In contrast, horizontal tubular PBRs, consisting of interconnected tubes, require high energy input due to continuous culture circulation and heat management. However, the integration of degassing systems helps regulating oxygen levels, improving microalgal growth efficiency. PBRs provide advantages such as reduced contamination risk, higher photosynthetic efficiency, and controlled environmental conditions. Despite higher operational costs, their ability to enhance biomass production and CO₂ fixation makes them a promising technology for large-scale microalgae cultivation and sustainable bioproduct generation.

The design of PBRs plays a crucial role in enhancing microalgae cultivation by optimizing mixing efficiency, light exposure, and gas–liquid mass transfer. PBRs rely on aeration for mixing, which requires less energy and fewer materials compared to pump-driven methods. However, challenges such as higher material costs, gas hold-ups, and biofouling, where microalgae adhere to the reactor walls thus blocking light penetration can impact efficiency. Kubar et al. developed the Tesla-valve and Nested-bottled PBRs, which generated internal vortices to improve microalgae suspension, leading to biomass production increases of 28.1% and 35%, respectively [26, 27]. Xue et al. (2013) introduced a 130 L air-lift flat-plate PBR equipped with optical fibers for internal illumination, enhancing *Spirulina platensis* growth [28]. Raeisossadati et al. [29] incorporated red and blue luminescent solar concentrators (LSCs) into raceway ponds, significantly improving *Arthrospira platensis* biomass productivity by 26% and boosting phycocyanin production by 44% [29]. Wang et al. (2023) developed a hybrid thin-layer fountain PBR (TLF-PBR) with a 1.5 m diameter, achieving a maximum biomass concentration of 3.955 ± 0.037 g/L 71% higher than flat-plate PBRs and 44.7% greater than thin-layer cascade systems [30]. Structural optimizations, such as adding internal static baffles and mixers, have been explored to improve mixing patterns and light distribution in flat-plate PBRs [31, 32]. Yaqoubnejad et al. [33] reported that integrating mechanical diversions and inclined baffles with airlift technology enhanced the flashing light effect, increasing biomass yield by 43.6% and 107.4% compared to standard PBRs [33]. Additionally, Cheng et al. [34] introduced an up–down chute baffle in a raceway pond, generating multiple internal vortices to enhance mixing [34]. A comprehensive understanding of multiphase flow, energy and mass transfer, and multi-field coupling is essential for developing highly efficient PBRs, enabling improved productivity and sustainability in microalgae-based bioprocesses [27].

Traditional PBRs often face limitations such as poor light penetration and inadequate mixing. This study presents the Nested-bottled Photobioreactor (NB-PBR) hybrid system, which eliminates these issues by employing vortex-driven mixing, enhancing light distribution and CO₂ dissolution. The continuous circulation between the raceway pond and NB-PBR optimizes nutrient availability, boosting microalgal growth and productivity. Despite similar energy and material requirements, the NB-PBR offers superior efficiency, lower energy consumption, and reduced operational costs, making it a cost-effective alternative to traditional PBRs.

## Material and methods

### Experimental setup for hybrid system

**Fig. 1:**
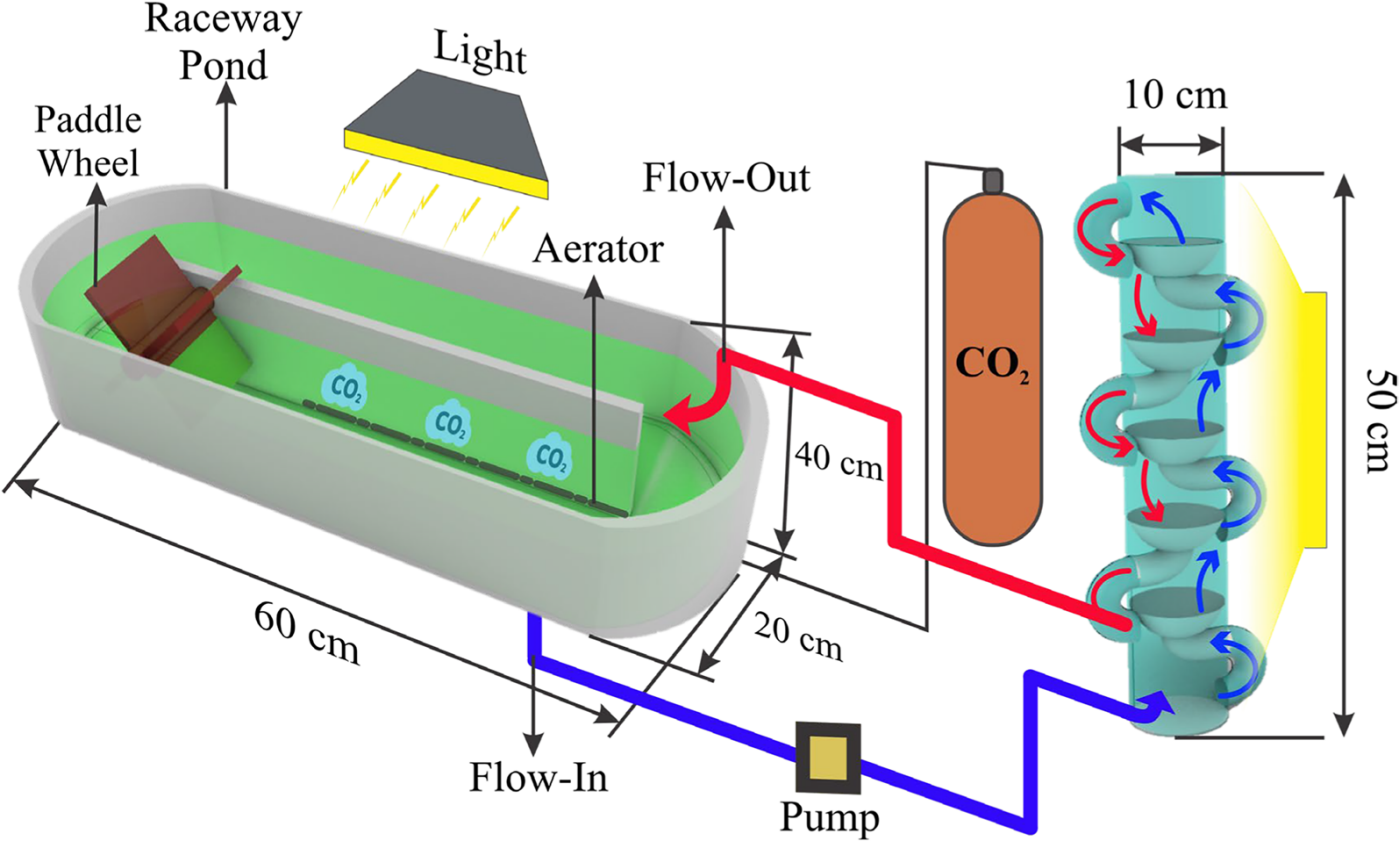
Schematic of the Hybrid Nested-bottled photobioreactor

In this experiment, two open raceway ponds were connected separately to a traditional PBR (vertical tubular PBR loop of equal volume integrated with the raceway pond, used as a control) and a novel NB-PBR, as illustrated in Fig. 1. The NB-PBR was designed and integrated with a raceway pond (20 cm wide, 60 cm long) to enable continuous medium circulation between the two systems. This novel PBR featured two interconnected columns with curved arms on both sides, promoting additional vortices within the solution to enhance system performance. Each column (50 cm in height, 10 cm in diameter) was divided into riser and downcomer sections, consisting of three regions, each 8 cm in height. Aeration was introduced from the bottom right to optimize gas–liquid interactions. The NB-PBR's innovative design facilitated effective modification of the solution’s flow path and improved CO₂ bubble distribution, enhancing overall efficiency. The hybrid system, operated under standard conditions of 12,000 ± 200 lx light intensity, 27.2 °C temperature, and 15% CO₂, provided optimal illumination to support the photosynthetic activity of *Arthrospira platensis* throughout its circulation in the system.

### Cultivation of *Arthrospira platensis* strain

A mutated strain of *Arthrospira platensis*, designated as *Spirulina* sp. ZJU9000, was cultivated in Zarrouk's medium. The mutation was induced using γ-rays from Cobalt-60 nuclear irradiation, followed by domestication through exposure to ~ 15% CO_2_ concentrations for eight generations to ensure hereditary stability. The cultivation medium, prepared with reagents from Sinopharm Chemical Reagent (Shanghai, China), was Zarrouk’s medium, a widely used formulation for *A. platensis* cultivation. This approach aimed to enhance the strain’s adaptability to high CO₂ conditions, supporting its potential for improved growth and biomass production in controlled environments [35].

### Measurements of mixing time and the mass transfer

Mixing time and mass transfer coefficients were assessed using pH probes (InPro3253i/SG/120, Mettler Toledo). In the open raceway pond (ORWP), five liters of distilled water were placed, and the initial pH was adjusted to 3.0 using 35% hydrochloric acid (HCl). Sodium hydroxide (NaOH) (2.5–3.5 mL, 12 µmol/L) was then added incrementally, producing two consecutive pH peaks, with the time interval between them recorded as the mixing time. For mass transfer evaluation, purified water was aerated with air and nitrogen (N₂), with gas flow precisely controlled by a mass flow meter (SevenstarCS200, China). The dissolved oxygen concentration was maintained between 4 and 6 mg/L by reducing it to ≤ 4 mg/L with nitrogen, followed by reoxygenation with ambient air. Changes in pH and oxygen levels were recorded every 0.1 s using pH and dissolved oxygen probes (Mettler Toledo) connected to transmitters (i-7017fc, ICP DAS, China) and monitored via data acquisition software [36]. Two sets of measurements were taken for both mixing time and mass transfer coefficient values, with the calculated mean and standard deviation reported.

### Measurement of photosynthetic parameters during *Arthrospira Platensis* cultivation

The maximum photochemical efficiency (F_v_/F_m_) of φPSII was determined utilizing the formula: F_v_/F_m_ = (F_m_–F_0_)/Fm, where F_m_ and F_o_ denote the maximal and minimal chlorophyll fluorescence yields of a sample subjected to dark adaptation. For these measurements, 2.8 mL of algal samples underwent a dark-adaptation period in a liquid sample holder for 15 min. The measurements were conducted using an AquaPen.

A 5-ml microalgal solution sample was obtained for chlorophyll a measurement. Subsequently, the sample were centrifuged at 8000 rpm for 5 min, and the supernatant was removed. Following this, 5 ml of methanol were introduced to the microalgal pellet, and the mixture was kept in darkness for 30 min after vortex mixing. The supernatant was then transferred into a cuvette. Absorbance at OD_665_ and OD_652_ was measured using pure methanol as the blank sample with a UV-3200B spectrophotometer. The overall chlorophyll content was calculated using the following formula [26].

1 $${\text{Chl}}\, - \,{\text{a}}\,{\text{mg}}/{\text{L}}\, = \,\left( {{16}.{5169}\, \times \,{\text{A}}_{{{665}}} \, - \,{8}.0{962}\, \times \,{\text{A}}_{{{652}}} } \right)$$

Sampling was done twice a day at 09:00 and 21:00.

### Measurement of phycocyanin and allophycocyanin

To extract allophycocyanin and phycocyanin, the culture was centrifuged at 4000 × g for 5 min. The biomass was then separated from the supernatant and homogenized for 2 min with 5 mL of a 1% CaCl₂ (calcium chloride) solution. The homogenized samples underwent three freeze–thaw cycles, followed by overnight incubation at 4 °C. After incubation, the samples were centrifuged at 8000 × g for 12 min, and the absorbance of the supernatant was measured using a spectrophotometer at 652 nm for allophycocyanin and 620 nm for phycocyanin [37].

2 $${\text{Phycocyanin}}\,\left( {{\text{mg}}/{\text{L}}} \right)\, = \,{\text{A}}_{{{62}0}} \, - \,0.{474}\, \times \,{\text{A}}_{{{652}}} /{5}.{34}$$

3 $${\text{Allophycocyanin}}\,\left( {{\text{mg}}/{\text{L}}} \right)\, = \,{\text{A}}_{{{652}}} \, - \,0.{2}0{8}\, \times \,{\text{A}}_{{{62}0}} /{5}.0{9}$$

where A_620_ and A_650_ are the absorbance at 620 at 650 nm, for phycocyanin and allophycocyanin, respectively. Data was collected in triplicate (n = 3), with averages, and standard deviation.

### Biomass and CO_2_ fixation measurements of ***Arthrospira platensis***

The dry mass was measured gravimetrically. To determine the sample weight, a pre-weighed dry filter paper was used to filter a 10 mL sample (Vaccum pump VOP-200, Yiheng, Shanghai China). The filtered sample was then dried in an oven at 50 °C for three hours. The biomass was calculated by measuring the difference between the initial weight of the dry filter paper and its final weight after filtration and drying. The CO_2_ fixation rate was measured via the dry weight conversion method using following equation:

$$CO_{2} \,fixation\,\left( {gL^{ - 1} d^{ - 1} } \right)\, = \,\frac{{\left( {w_{2} \, - \,w_{1} } \right)\, \times \,P\, \times \,M_{CO2} }}{{\left( {t_{2} \, - \,t_{1} } \right)\, \times \,M_{c} }}$$

where w_2_ is the dry mass at time t_2_ and w_1_ is the biomass dry weight at time t_1_, p represents carbon content in microalgae cells (50%), M_CO2_ represents the molecular weight of CO_2_, and M_C_ denotes the molecular weight of carbon [38, 39].

## Results and discussion

### Effects of traditional and Nested-bottled PBR on MTC and MT

The PBR’s mass transfer capacity (MTC) relates to its proficiency in mass transfer, while the mixing capacity is quantified by the mixing time (MT). In general, microalgae growth is typically increased in the presence of CO_2_ compared to air. Furthermore, both structural factors and CO_2_ significantly influence the overall performance of a well-designed column PBR. Earlier studies indicate that the introduction of circular or curved baffles in PBRs and ORWPs generates a vortex flow field, demonstrating its effectiveness in enhancing microalgae biomass productivity [38, 39].

**Fig. 2:**
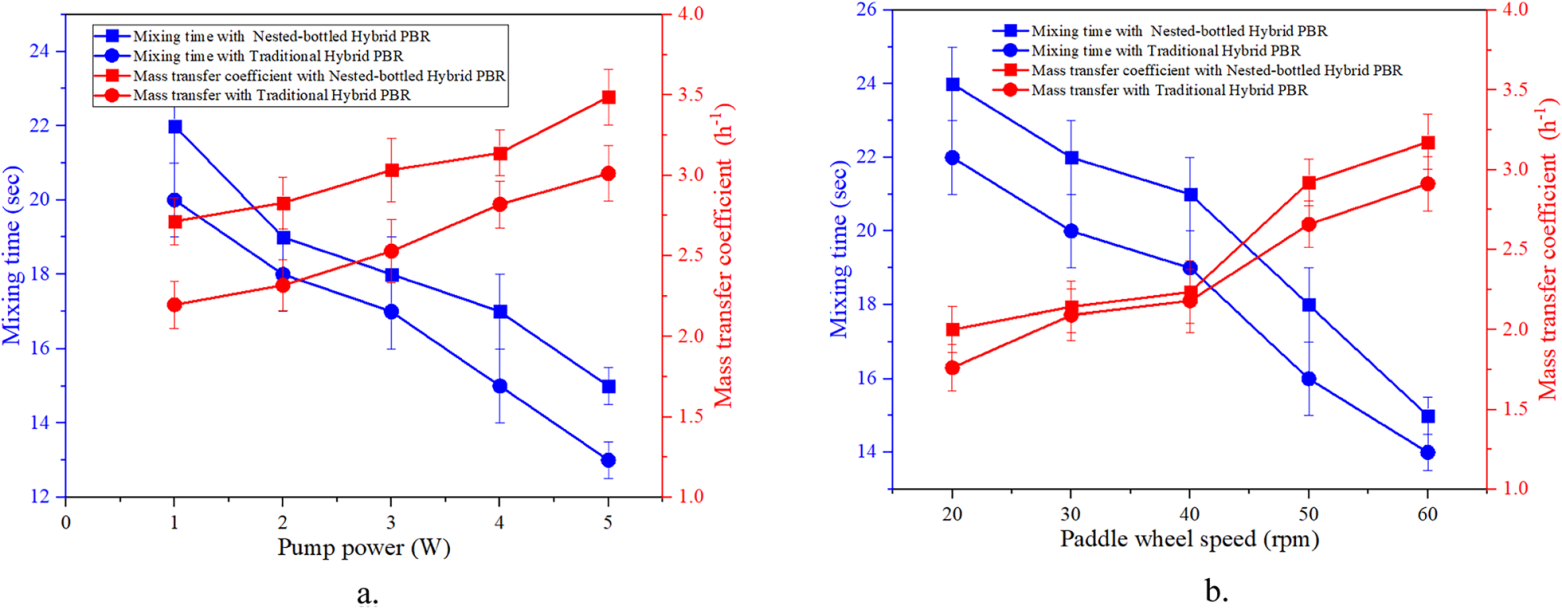
Mixing time (MT) and mass transfer coefficient (MTC) for traditional and Nested-bottled hybrid PBR. **a** Effect of pump power **b** Effect of paddle wheel speed

The degree of liquid-phase homogenization in the PBR or ORWP indicates the efficiency with which various nutrients and dissolved CO_2_ achieve a uniform status within the microalgal suspension [40]. In an ORWP, the primary location for the mixing process is around the paddle wheel. After the utilization of inorganic carbon and nutrients through photosynthesis in the raceway pond, the microalgal suspension is circulated to the NB-PBR. The addition of a nested structure reduced MT and increased mass transfer as compared to the traditional PBR (T-PBR) hybrid system (Fig. 2). The flow field in the NB-PBR hybrid system is the primary factor that determines the inner mixing ability. The flow of the microalgal suspension is directed along specific sections to extend the flow pathway, enhancing the mixing and interaction of microalgal suspensions. This occurs not only from the bottom to the top of the NB-PBR but also from the central regions to the outer regions. In contrast, the T-PBR and open raceway pond demonstrate lower mixing efficiency as it contains larger inactive areas, where substance exchange depends on diffusion, leading to a considerable slowdown in the mixing process. The evaluation of the influence of the NB-PBR on mixing time was conducted in this study when connected together with an OWRP. The presence of curved structures in the NB-PBR resulted in notable flow resistance, impeding the mixing process. With an escalation in pump power from 1 to 5 W, the mixing time decreased from 22 to 15 seconds (Fig. 2a). Increased pump power accelerated the circulation of the microalgal suspension, thereby enhancing flow intensity and significantly reducing mixing time.

For the NB-PBR hybrid system, MT decreased from 24 to 15 s however the mass transfer coefficient increased from 1.95 to 3.23 h^−1^ when the rotation speed increased from 20 to 60 rpm (Fig. 2b). The decrease in MT signifies the even distribution of nutrients within the medium. During the microalgal growth phase, the regions with higher light intensity exhibited an elevated growth rate, consequently accelerating the rate of nutrient consumption. The NB-PBR hybrid system was partitioned into two segments: the riser and the downcomer, aimed to maintain uniformity throughout the culture, optimizing even distribution of light penetration across the PBR. The proposed design of the NB-PBR hybrid system enhanced the light absorption capacity of microalgae cells by facilitating frequent movement between light and dark zones. This strategy prevented prolonged exposure of cells to either zone, mitigating the risk of photoinhibition and respiratory issues, ultimately contributed to reduce *A. platensis* growth. Additionally, the NB-PBR hybrid system improved intracellular energy supply and facilitated processes such as NADH oxidation, transmembrane protein transport, oxidative phosphorylation, ATP synthase, and proton gradient formation [41, 42].

### Photochemical efficiency properties for traditional and Nested-bottled PBR

In the NB-PBR hybrid system, as the liquid traversed the inner tubes and entered the primary column sections, vertical vortex flow fields in both clockwise and anticlockwise direction are induced. The generation of these vortices distinguishes the NB-PBR hybrid system from T-PBR designs that necessitate modifications for similar flow patterns. Unlike T-PBRs, the NB-PBR was independent of inner and outer zones for microalgae solution circulation, choosing instead to divide the entire flow field with a riser and down-flow zone. In the T-PBR hybrid system, the fluid exhibited a laminar pattern in the inner up-flow area and the outer down-flow area.

The installation of the NB-PBR hybrid system significantly improved the flow dynamics conditions. This innovative design of the NB-PBR hybrid system has a substantial impact on the advancement in algal cultivation technology. *A. platensis* exhibits a notable CO_2_ fixation rate, substantial protein content, and a rich pigment profile, particularly phycocyanin. The photosynthetic lamellae, forming thylakoids, house phycobilisomes—a specialized supramolecular pigment protein complex [44]. These phycobilisomes function as light-harvesting antennae, binding chromophores like phycocyanobilin. These antennae systems capture light energy from spectral regions, where chlorophyll a either weakly absorbs or does not absorb at all, subsequently transferring the absorbed energy to the reaction center. Environmental factors influence phycobilisome content, leading to variations [45]. Elevated phycobilisome content enhances photosynthetic efficiency, contributing to a substantial 28.7% increase in biomass yield in NB-PBR hybrid system. The role of chlorophyll within this inner antenna involves photon capture and the reception of excitation energy from the phycobilisome, directing it to chlorophyll in the reaction center where primary photochemistry occurs [46]. The light-harvesting pigment protein complex of *A. platensis* encompasses phycobilisomes, comprising allophycocyanin (APC) and phycocyanin (PC) [47].

**Fig. 3:**
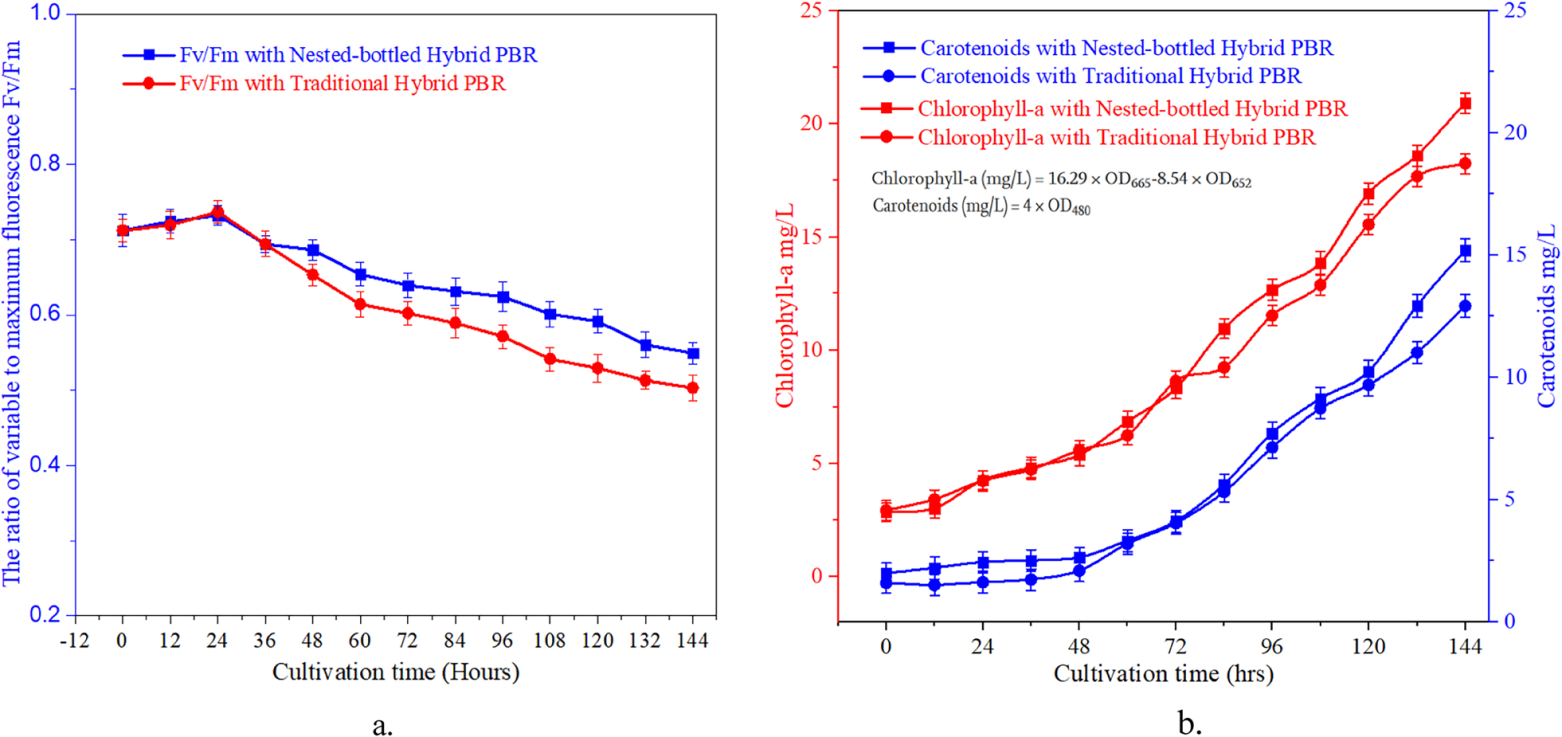
Comparison of chlorophyll fluorescence induction kinetic parameters of *A. platensis* cells in the traditional and Nested-bottled hybrid PBR **a** Fv/Fm **b** Chlorophyll-a and carotenoids

The fluorescence parameter Fv/Fm stands out as the most assessed measurement for estimating the PSII system's activity. It serves as a crucial indicator of photosynthetic efficiency and the conversion of PSII reactions. As depicted in Fig. 3a, the Hybrid-PBR demonstrated elevated Fv/Fm 8.7%, attributed to the lower microalgal density facilitating improved light penetration throughout the PBR. Consequently, the maximum photochemical efficiency and absorbed light energy of the reaction center increased, enhancing the likelihood of algal cells absorbing a greater number of light photons. With the progression of biomass growth, a decline in photosynthetic efficiency occurred, resulting in a substantial reduction in the percentage of light deficiency or photodamage experienced by algal cells. Simultaneously, efficient mixing and mass transfer enhanced the overall performance of the NB-PBR hybrid system. The even distribution of algal cells throughout the NB-PBR hybrid system contributed to increased biomass production efficiency per absorbed photon. The structural modification applied to the NB-PBR hybrid system had a favorable impact on F_v_/F_m_, closely associated with light utilization efficiency.

The role of chlorophyll within this inner antenna is dual—capturing photons directly and receiving excitation energy from the phycobilisomes, subsequently directing it to chlorophyll in the reaction center where primary photochemistry occurs [17]. This signifies the intactness of the cells' photosynthetic system, with enhanced photosynthesis. Carotenoids play a crucial role in antenna systems and chlorophyll binding proteins, aiding in light energy capture and the removal of excess free radicals in microalgae cells. In *A. platensis*, chlorophyll is chlorophyll-a. The photosynthetic efficiency of the cell is directly proportional to the content of chlorophyll-a, elevated chlorophyll-a and carotenoid content in NB-PBR hybrid system (Fig. 3b) showed positive influence over photosynthesis and CO_2_ fixation in *A. platensis *cells. The increased carotenoid content also strengthened stress resistance, including resistance to light damage and free radical accumulation. *A. platensis* reproduces through fission, where one trichome divides into two or more new individuals of the same size and shape.

### Phycocyanin and Allophycocyanin content in hybrid NB-PBR and hybrid T-PBR

**Fig. 4:**
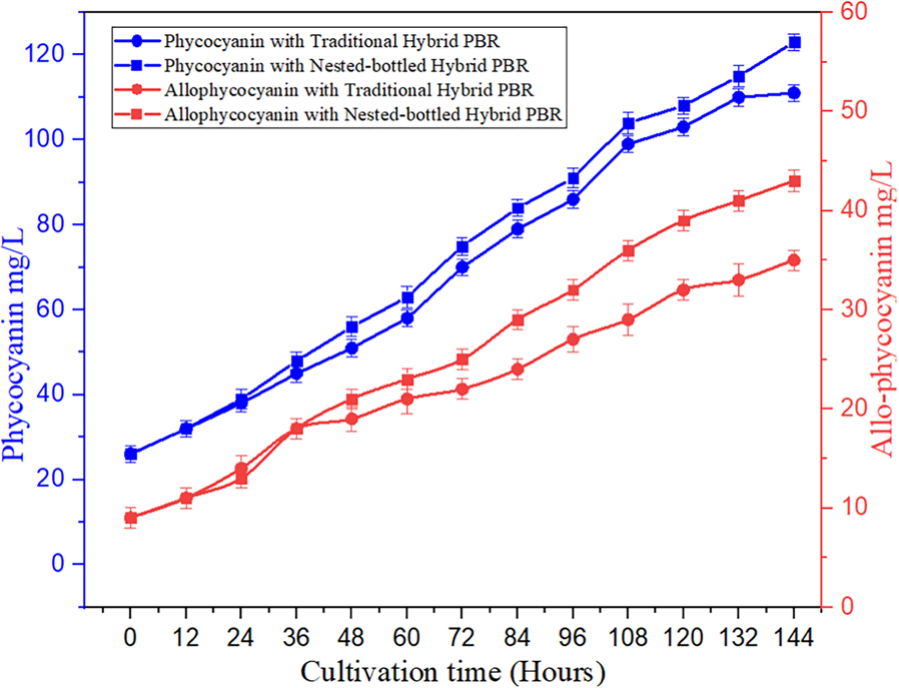
Phycocyanin and Allophycocyanin

The comparative investigation of pigment composition in *A. platensis* cultivated within the NB-PBR hybrid system and the Hybrid T-PBR revealed significant differences shown in Fig. 4, shedding light on the influence of reactor configuration on pigment levels. C-phycocyanin stands out among high-value proteins due to its antioxidant and anti-inflammatory properties [48]. The study demonstrates the significant positive influence of the hybrid PBR on the growth of *A. platensis*. The light-harvesting pigment-protein complex in *A. platensis* comprises phycobilisomes, consisting of allophycocyanin and phycocyanin [49]. Over a 144-h cultivation period, *A.platensis* grown in the NB-PBR hybrid system exhibited notably higher concentrations of both allophycocyanin and phycocyanin compared to those in the Hybrid T-PBR. Specifically, the T-PBR hybrid system yielded the reduced phycocyanin concentration at 110 mg/L, while the NB-PBR hybrid system recorded the highest at 123 mg/L, indicating a considerable impact of reactor type on phycocyanin production (Fig. 4). Similar trends were observed for allophycocyanin, with the NB-PBR hybrid system demonstrating superior pigment synthesis capabilities compared to the T-PBR hybrid system 43 and 35 mg/L, respectively. These findings highlight the importance of reactor design in influencing pigment production, thereby affecting the nutritional quality and commercial viability of cultivated *A. platensis*.

The superior pigment synthesis environment provided by the NB-PBR hybrid system suggests its potential as a preferred system for maximizing valuable pigment production in *A. platensis* cultivation. This nuanced understanding of pigment dynamics contributes valuable insights to the optimization of microalgal cultivation strategies, ultimately enhancing both nutritional and economic outcomes [50].

b. b

### Hybrid NB -PBR increased CO_2_ fixation biomass dry weight

**Fig. 5:**
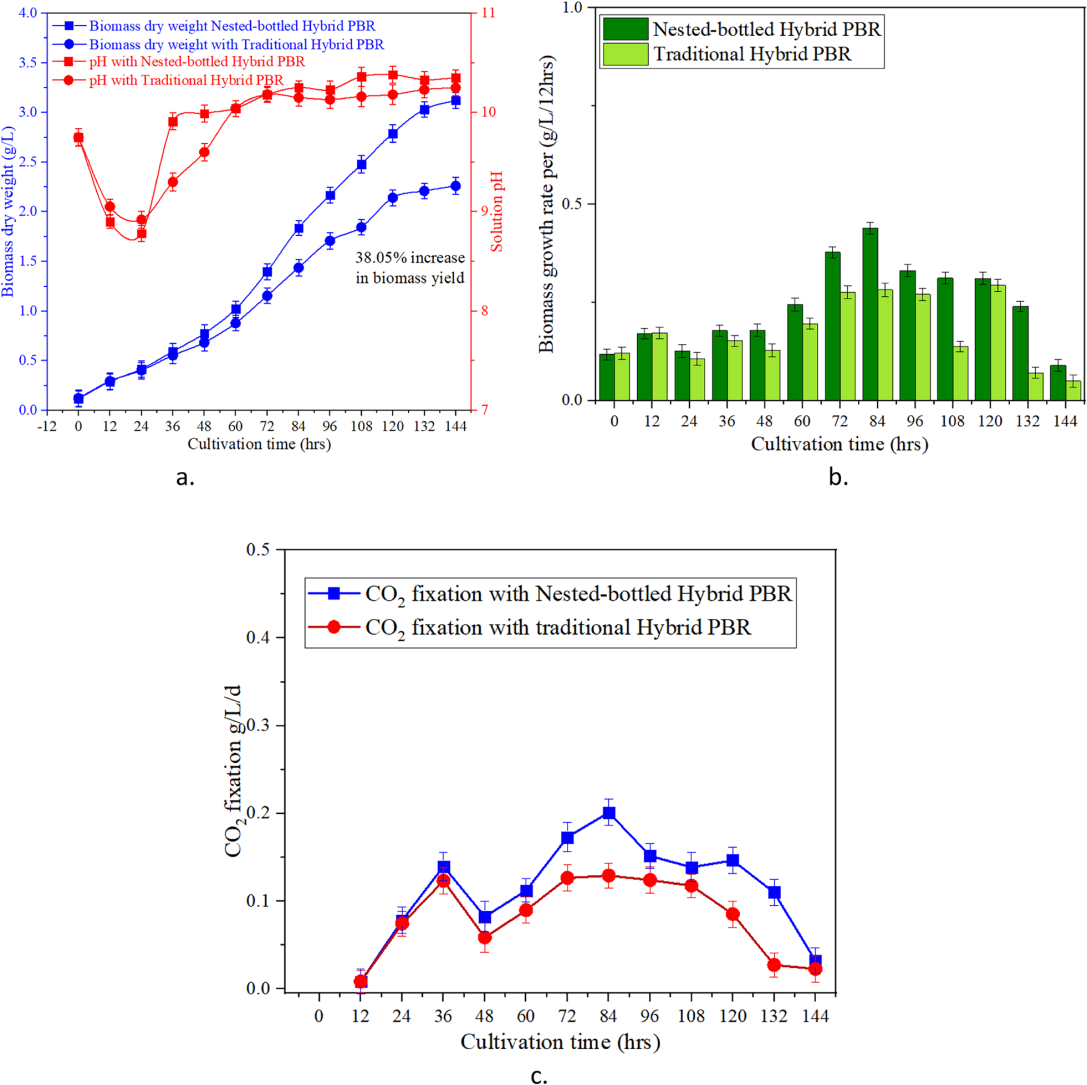
**a** *A. platensis* dry mass and pH value with 15% CO_2_ [where “ρ” is the carbon content in microalgae dry weight (50%)], **b** Rate of growth per 12 h, **c** CO_2_ fixation rate

PBRs under optimal conditions, entering the exponential growth phase on the second day in both reactors. As the microalgae continued to proliferate, the concentration in the photobioreactors increased, necessitating elevated dissolved inorganic carbon and well-mixed nutrients to sustain the rapid growth rate. By the end of the 144-h cultivation period, the final microalgal biomass accumulation reached 38.0% rise in dry mass (3.1 g/L) and 2.26 g/L in the hybrid NB-PBR and T-PBR hybrid system, respectively (Fig. 5). This improvement was attributed to the enhanced mass transfer coefficient and improved mixing state facilitated by the NB-PBR. Upon initial aeration of CO_2_ into the PBR, the pH of the microalgal suspension promptly decreased from 9.8 to 8.9, a result of the carbon dioxide hydration process (CO_2_ + H_2_O → HCO_3_^–^ + H^+^)[27, 51]. After 12 h, the pH gradually increased due to the consumption of nitrite and dissolved inorganic carbon nutrients. After 36 h, the pH exhibited further variation as the microalgal concentration significantly increased (Fig. 5a), leading to accelerated nutrient consumption and an ensuing increase in pH. In summary, the NB-PBR offers a viable strategy for optimizing solar energy utilization and enhancing microalgal biomass yield as compared to T-PBR hybrid system.

The specific design and construction of the NB-PBR were geared towards generating predominant vortices within the reactor, ensuring an even distribution of nutrients, and preventing cell adhesion to the PBR walls. This distinctive structure also extends the duration of gas–liquid contact and facilitates the dissolution of CO_2_. The movement of CO_2_ bubbles through the PBR induces liquid flow, and each segment of the NB-PBR temporarily retains the bubbles before releasing them into the subsequent section. This sequential process results in the breakdown of larger bubbles into smaller ones, enhancing gas–liquid mass transfer and prolonging the retention of smaller bubbles within the PBR. Consequently, there is an increased CO_2_ mass transfer (Fig. 5c). The adoption of microbubbles, as opposed to larger bubbles, significantly enhances the efficiency of CO_2_ absorption.

## Conclusion

Over 144 h of *Arthrospira platensis* cultivation, integrating an ORWP with a NB-PBR significantly enhanced CO₂ mixing and mass transfer, promoting microalgal growth. Continuous culture circulation optimized light exposure and carbon utilization, increasing photosynthetic efficiency (Fv/Fm) by 8.7%. This led to a 38.0% rise in dry mass (3.1 g/L), with mass transfer and mixing efficiency improving by 16.6% and 15.3%, respectively. Enhanced gas–liquid dissolution further supported growth and CO₂ fixation. These findings demonstrate the potential of NB-PBR hybrid system as an efficient and sustainable approach for large-scale *A. platensis* cultivation.

## Data Availability

No datasets were generated or analysed during the current study.
